# Supplementary material for: Chronic postnatal chemogenetic activation of forebrain excitatory neurons evokes persistent changes in mood behavior
Source: eLife. 2020 Sep 21;9:e56171. doi: 10.7554/eLife.56171 (PMC7652419; doi:10.7554/eLife.56171)
Supplement: Transparent reporting form [file elife-56171-transrepform.pdf]

eLIFE
1st Floor
24 Hills Road
Cambridge CB2 1JP, UK
P 01223 855340
W elifesciences.org
T @elife

eLife's transparent reporting form

We encourage authors to provide detailed information *within their submission* to facilitate the interpretation and replication of experiments. Authors can upload supporting documentation to indicate the use of appropriate reporting guidelines for health-related research (see EQUATOR Network), life science research (see the BioSharing Information Resource), or the ARRIVE guidelines for reporting work involving animal research. Where applicable, authors should refer to any relevant reporting standards documents in this form.

If you have any questions, please consult our Journal Policies and/or contact us: editorial@elifesciences.org.

Sample-size estimation

- You should state whether an appropriate sample size was computed when the study was being designed
- You should state the statistical method of sample size computation and any required assumptions
- If no explicit power analysis was used, you should describe how you decided what sample (replicate) size (number) to use

Please outline where this information can be found within the submission (e.g., sections or figure legends), or explain why this information doesn't apply to your submission:

An appropriate sample size was used for each experiment, as standardized and described in available literature. No explicit power analysis was done to compute sample size. First of all, we did not make any a priori assumption about either the direction or the size of effects when we designed the experiment or performed random assignments. While we are aware that a bigger sample size would have ensured a reduction in Type-I error (which could occur due to lower sample size, variation, or both), we would have needed > 30 bigenic animals per group to achieve a statistical power of 0.9 or above considering the size of effect. It is a technical and logistic limitation for the kind of experiments performed, as generating the desired number of bigenic animals would be extremely difficult. However, we have performed multiple behavioural assays when possible in order to arrive at our conclusions.

Replicates

- You should report how often each experiment was performed
- You should include a definition of biological versus technical replication
- The data obtained should be provided and sufficient information should be provided to indicate the number of independent biological and/or technical replicates
- If you encountered any outliers, you should describe how these were handled
- Criteria for exclusion/inclusion of data should be clearly stated
- High-throughput sequence data should be uploaded before submission, with a private link for reviewers provided (these are available from both GEO and ArrayExpress)

Please outline where this information can be found within the submission (e.g., sections or figure legends), or explain why this information doesn't apply to your submission:

The required information can be found in Methods and Figure legends. Sample-size used for each experiment is described in the figure legends of the respective figure. Throughout the manuscript, 'n' is used to indicate biological replicates.
We have not excluded any animals from the data analysis in all the experiments represented in our results, with the exception for the PNCNO behavioural cohort for anxiety-like behaviour tests. When performing anxiety-like behaviour tests for the PNCNO treated males, we removed one outlier each from the Vehicle and PNCNO groups as they were more than three standard deviations away from the entire cohort on the LD avoidance test, and were outliers by the Grubb's test. These animals were removed from all anxiety-like behavioural assay data.

Statistical reporting

- Statistical analysis methods should be described and justified
- Raw data should be presented in figures whenever informative to do so (typically when N per group is less than 10)
- For each experiment, you should identify the statistical tests used, exact values of N, definitions of center, methods of multiple test correction, and dispersion and precision measures (e.g., mean, median, SD, SEM, confidence intervals; and, for the major substantive results, a measure of effect size (e.g., Pearson's r, Cohen's d)
- Report exact p-values wherever possible alongside the summary statistics and 95% confidence intervals. These should be reported for all key questions and not only when the p-value is less than 0.05.

Please outline where this information can be found within the submission (e.g., sections or figure legends), or explain why this information doesn't apply to your submission:

The desired information on statistical analysis is available in the manuscript under the section 'Materials and Methods'. For each experiment, mean ± S.E.M. as precision measure and respective *p*-values have been stated for each measure in both the 'Result' section as well as in the figure legends.
An entire summary of statistical analysis has been provided as metadata in supplementary information.

(For large datasets, or papers with a very large number of statistical tests, you may upload a single table file with tests, Ns, etc., with reference to sections in the manuscript.)

Group allocation

- Indicate how samples were allocated into experimental groups (in the case of clinical studies, please specify allocation to treatment method); if randomization was used, please also state if restricted randomization was applied
- Indicate if masking was used during group allocation, data collection and/or data analysis

Please outline where this information can be found within the submission (e.g., sections or figure legends), or explain why this information doesn't apply to your submission:

All experiments were performed in two groups. The animals were 'randomly assigned' to either CNO or vehicle-treated group. An entire litter was either treated with CNO or with vehicle from postnatal day 2 to postnatal day 14. For juvenile/ adult experiments, animals were randomly assigned to a group, segregated into cages, and treated with only either CNO or vehicle per cage. For all experiments, animals from at least three litters were included in a group in order to avoid any litter-related bias. No masking was used during either group allocation, data collection/ analysis.

Additional data files ("source data")

- We encourage you to upload relevant additional data files, such as numerical data that are represented as a graph in a figure, or as a summary table
- Where provided, these should be in the most useful format, and they can be uploaded as "Source data" files linked to a main figure or table
- Include model definition files including the full list of parameters used
- Include code used for data analysis (e.g., R, MatLab)
- Avoid stating that data files are "available upon request"

Please indicate the figures or tables for which source data files have been provided:

We have made the source data available for Figure 2 and Figure 3.
